# EFFICACY OF A SINGLE SESSION OF ANTICIPATORY POSTURAL ADJUSTMENTS TRAINING TO SUPPORT PEOPLE WITH PARKINSON’S OVERCOMING FREEZING OF GAIT: A MULTI-METHODS APPROACH

**DOI:** 10.2340/jrm.v57.42491

**Published:** 2025-05-16

**Authors:** Yuri RUSSO, Zijing WANG, Jiaxi YE, Phaedra LEVERIDGE, Alice NIEUWBOER, Mark WILSON, Meriel NORRIS, Elmar KAL, Sarah E. LAMB, William R. YOUNG

**Affiliations:** 1 University of Exeter, Exeter, UK; 2 Katholieke Universiteit Leuven, Leuven, Belgium; 3 Brunel University London, London, UK

**Keywords:** Parkinson’s disease, freezing of gait, step initiation, anticipatory postural adjustment, weight-shifting, festination, virtual reality

## Abstract

**Objective:**

To assess the efficacy of anticipatory postural adjustments training on the ability to successfully step from freezing of gait, and to evaluate the contribution of attentional processes to potential benefits using an additional attentional-control training intervention.

**Design:**

Crossover-design.

**Subjects/Patients:**

Nineteen people with Parkinson’s and freezing (females: 10; age:75.5 ± 7.5 years) tested while ON medication.

**Methods:**

Participants navigated a cluttered virtual domestic environment with freeze-provoking tasks. Assessments occurred in the laboratory at baseline, post-anticipatory postural adjustments training, and post-attentional-control training, with randomized training order. All training was video-based. Video annotation was used to identify freezing events. Participants’ immediately recollected thoughts they had during the tasks were analysed with content analysis. Perceived safety and effectiveness of the strategies were reported in follow-up calls held 4 weeks post-assessment.

**Results:**

Successful step initiations increased from 57% at baseline to 77% post-anticipatory postural adjustments training (*p* = 0.034). Participants rated the interventions as safe and effective, reporting increased balance confidence (70% to 90%), and reduced fear (*p* = 0.01), after the anticipatory postural training. Attentional-control training alone was perceived as less effective compared with more goal-directed anticipatory postural adjustments training.

**Conclusion:**

Video-based anticipatory postural adjustments training significantly improved step initiation from freezing when used during challenging tasks and in complex environments. Anticipatory postural adjustments training shows promise as an effective “rescue strategy” that could be learned remotely/at home.

## LAY ABSTRACT

Freezing of gait is a common and disabling symptom of Parkinson’s disease, where people describe the sensation of their feet being glued to the floor. This often leads to falls, anxiety, and reduced quality of life. Our study explored whether a single session of video-based training (anticipatory postural adjustments training), focused on weight-shifting, could support people with Parkinson’s to successfully initiate steps from freezing episodes. Nineteen participants navigated virtual environments with tasks designed to provoke freezing. Their stepping movements and thoughts were recorded before and after the anticipatory postural adjustments training. The results showed significant improvements in step initiation from freezing after the anticipatory postural adjustments training, with participants also reporting increased confidence and reduced fear. We also compared anticipatory postural adjustments training with another video-based intervention targeting attentional control, which participants found to be less effective. Our findings suggest that the anticipatory postural adjustments training could offer a simple, cost-effective way to help people with Parkinson’s manage freezing in their daily lives.

More than 50% of people with Parkinson’s disease (pwPD) experience freezing of gait (FoG), defined as a “brief, episodic absence or marked reduction of forwarding progression of the feet despite the intention to walk” (1). FoG is one of the most debilitating symptoms of PD and is associated with falls, anxiety, and reduced quality of life (1, 2). FoG is mostly managed by optimizing pharmacological and surgical interventions. However, limitations in the efficacy of these treatments emphasize a need to improve approaches to physical therapy and self-management of FoG (3). For this reason, compensatory strategies, such as sensory cues, are often used to improve gait characteristics and reduce/prevent FoG (4). However, when FoG occurs, people need effective strategies to safely initiate walking. Indeed, it is within freeze events that people are at greatest risk of falling as they try to force a step (5) and particularly vulnerable to anxieties known to further exacerbate freezing (6).

When initiating a step, people typically require anticipatory postural adjustments (APA), involving a weight-shift towards the non-stepping leg followed by forward propulsion (7). Defective APA are often observed in pwPD and FoG (8, 9), even when ON medication (10, 11). These changes in APA characteristics are thought to contribute to failed gait initiation and therefore represent a promising target for intervention (1, 12). Previous studies show that training pwPD and FoG to consciously produce an APA improves the initiation of forward (13) and turning steps (14), thereby highlighting APA-specific training as a simple and low-cost “rescue” strategy for use in daily life. However, these studies were conducted in controlled laboratory settings with constrained tasks (i.e., stepping/turning in place), limiting their generalizability to the complex demands of real-world environments. They also relied on in-person training with movement specialists (13), which may be a further limitation in community settings where access to specialists is limited. The COVID-19 pandemic further highlighted the need for effective remote support and self-management (15), raising the potential of delivering this training through a pre-recorded video for eventual independent home use.

It is necessary to understand whether: (*i*) previous positive results translate to more complex and dynamic tasks; (*ii*) the learned strategy can be used safely. We therefore co-designed and developed video-based APA training with our Patient and Public Involvement (PPI) group. Our primary aim was to test whether this training is safe to use and effective in improving successful stepping from FoG and reducing freezing time. We predicted that participants would perceive the strategy as safe and demonstrate improvements in successfully stepping from FoG (reducing time spent freezing) compared with pre-training conditions, and 4 weeks after the laboratory visit (self-assessment only).

The potential benefits of APA training likely arise from a combination of biomechanical factors (i.e., APA characteristics), enhanced goal-directed attention, and reduced anxieties (16, 17). Anxiety is known to increase attentional demands of gait by diverting attention to task-irrelevant stimuli (17, 18), leading to inefficiencies that likely exacerbate FoG (16). To explore these inefficiencies we developed a complementary video-based attentional-control (AC) training designed to target these potentially maladaptive processes. This arm of the study aimed to explore whether AC training improved self-reported confidence in balance and stepping from FoG, and whether combining the interventions (APA+AC training) provided additional benefits.

## Methods

### Study design

The study protocol was pre-registered on Open Science Framework (osf.io/7mxfp). All participants were tested ON medication (~60 min after intake of their regular dopaminergic medication) on the same day. To ensure participants remained in their ON medication state, their medication status was monitored throughout the session, with re-administration allowed if necessary. The study followed a crossover design where all participants, after baseline testing, were exposed to 2 interventions (i.e. APA training and AC training videos). The experimental protocol consisted of 3 test phases (*i*) baseline, (*ii*) post-APA training, and (*iii*) post-AC training. Each phase was separated by immediate retrospective recollection of thought processes followed by a rest and interaction with the subsequent training video. Within each test phase, participants completed a series of walking tasks while wearing a VR headset (HTC VIVE Pro-Eye II) showing a complex domestic scene (further details in Appendix S1). The sequence of the training videos was block randomized to either A-B-C or A-C-B (A = baseline, B = APA training, C = AC training).

### Participants

Twenty-four pwPD who experience FoG were recruited through the Parkinson’s UK research network. The inclusion criteria were: (*i*) a diagnosis of idiopathic PD (UK Brain Bank Criteria); (*ii*) recent experience of FoG, determined by the first item of the New Freezing of Gait Questionnaire (NFOGQ) (19)); (*iii*) able to walk unsupported for at least 1 min; (*iv*) over 55 years old. People were excluded if they had: (*i*) cognitive impairment (Montreal Cognitive Assessment Score (MOCA) < 21 [20]); (*ii*) any injury or disorder that may affect balance other than Parkinson’s; (*iii*) impaired normal or corrected to normal vision (Snellen Visual Acuity < 12/18). All participants provided written informed consent prior to the experiment. The study was approved by the local Institutional Review Board of the University of Exeter.

### Procedure

**Fig. 1:**
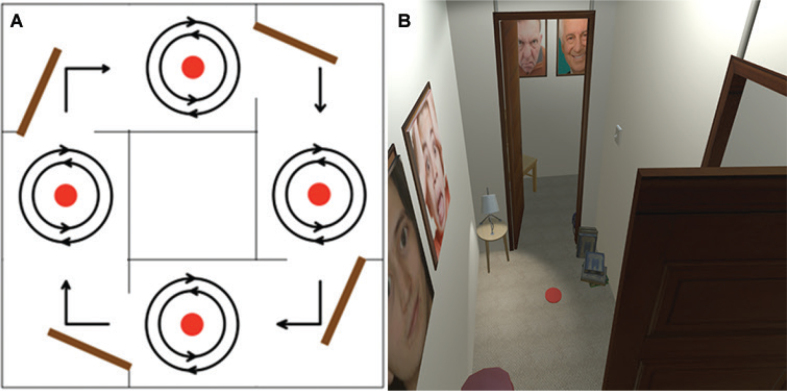
(A) A bird’s-eye perspective of the walking protocol. Participants navigate through virtual corridors (grey lines) with their paths depicted by black arrows. Red dots indicate designated stopping and turning points, while brown rectangles represent open narrow doorways. (B) Example of a virtual corridor from a participant’s perspective.

Participants attended a single testing session at the VSimulators laboratory, University of Exeter. First, participants were asked to perform a walking test where they followed markers on 3.7 by 3.7 m floorspace and familiarized themselves with tasks that would be used in subsequent tests (Fig. 1). Participants were then seated, a VR headset was fitted, and participants familiarized themselves with the virtual environment they would navigate during the next walking tasks. The VR was designed to reproduce common triggers of FoG (21), including cluttered corridors, requirements for turning actions and narrow doorways (Fig. 1). In the middle of each corridor there was a red circle, flush with the floor, representing a target on which people were asked to stop and turn 360˚ in clockwise and counterclockwise directions (22). Participants traversed a minimum of 6 doorways when navigating the corridors in both clockwise and anticlockwise directions (i.e., ≥ 12 corridors in -total). Participants were instructed to stop after completing 8 corridors in each direction. A detailed protocol is described in Appendix S1.

Immediately after completing each walking phase, participants were asked to sit down and rate (*i*) their level of confidence, on a scale from 1 to 100, in their ability to: (a) initiate walking from FoG on their first attempt, and (b) step out of FoG without falling; (*ii*) both their perceived levels of fear and any worrisome thoughts associated with stepping from a freeze or voluntary stopping, on an 11-point Likert scale^1^. Participants were also asked to provide an immediate recollection of any thoughts they remembered having during the walking task and during any FoG events experienced. Verbal descriptions were elicited by the same researcher (WY).

A follow-up video call was arranged 4 weeks after the testing session. For this 4-week period, participants did not have access to the training videos, nor were they specifically instructed to continue using any of the interventions. Outcomes from follow-up calls were intended to reflect any unprompted use of the learned strategies. During the call, participants reported on their use of the strategies in daily life (questions 1 and 3 of the follow-up questionnaire) and rated their perceived safety and effectiveness on a scale of 1 (lowest) to 11 (highest). All calls were conducted by the same researcher (YR). The list of questions used during the video call is provided in Appendix S1.

### Instructional videos

The APA and AC video content was co-designed by a Project Advisory Group (PAG) through an iterative process of evaluation and content refinement. We used storyboards, sketches for animations and draft videos which were evaluated by the PAG within: (*i*) group meetings, (*ii*) independently through anonymous online post-it note exercises (Google Jamboard), and (*iii*) individual meetings/emails with researchers.

The APA-training video was based on observations that APA are impaired in pwPD and FoG and that this impairment is one of the potential causes of failed gait initiation (13). The APA training emphasized the conscious production of appropriately scaled APA, aiming to shift participants’ control from the typically impaired automatic control of posture to a more deliberate control.

The AC training addressed the distractions and/or worrisome thoughts and cognitive inefficiencies known to trigger/exacerbate FoG. The training supported people in identifying and managing these thoughts, to break the FoG event. More information regarding the content of the videos is available in Appendix S1.

### Data analysis

FoG events were identified offline by a panel of 3 trained researchers using the camera recordings of the walking tasks. After identifying a FoG event, the onset and offset of each event were timestamped on the recording using ELAN software (23). Then, following a standardized annotation process (13, 14) each researcher made categorical judgements as to whether the first visually observable attempted step following FoG onset was either successful or unsuccessful^2^. In the case of disagreement among the panel, the event was classified as “pending” and reassessed later by a larger panel of researchers (YR, ZW, JY, PL, and WY). Only events where the panel reached unanimous agreement were included in the analysis. During the annotation process, researchers were blinded to the phase they were evaluating.

Participants’ characteristics (i.e., age, sex, BMI, MDS-UPDRS-III on medication) were recorded immediately before walking trials. Outcomes from video annotation were used to calculate: (*i*) percentage of successful steps following a FoG event: the number of successful steps/numbers of (successful + unsuccessful) x 100; (*ii*) number of FoG events; (*iii*) mean duration of FoG events, and (*iv*) percentage of time spent freezing (24).

### Sample size

The intervention was evaluated using a within-subject, pre- vs post-training crossover design with the primary comparison being between baseline vs post-APA training (arm 1 of the study). A sample size calculation (power 80%) was undertaken based on effect sizes reported by Maslivec and colleagues (13). We estimated that at least 6 participants experiencing FoG in both baseline and post-APA training phase (see phase 1 and 2 in arm 1 of the study) would be required to perform a one-tailed, related-samples Wilcoxon signed-rank test. As only ~50% of participants were expected to experience FoG while on medication, we required 24 participants in total (i.e., 12 in arm 1 of the study) to conduct statistical comparisons on FoG characteristics between baseline and post-APA training. As the efficacy of AC training and the efficacy of the combined interventions were part of secondary/exploratory analyses, the study was not powered to perform these comparisons.

### Statistical analysis

Statistical analysis was performed using SPSS (v.28, IBM Corp, Armonk, NY, USA). Non-parametric tests were used due to the small sample size. For all the analyses level of significance was set to α = 0.05.

A one-tailed Wilcoxon signed-rank test was used to compare the percentage of successful steps, FoG duration, FoG incidence, and reported confidence levels between baseline and post-APA training. We did not perform statistical comparisons for FoG characteristics in experimental phases where fewer than 6 participants experienced FoG.

Separate two-tailed Wilcoxon signed-rank tests were used to compare self-reported confidence, fear and worrisome thoughts between baseline and the 1st intervention, and between the 1st intervention and 2^nd^ intervention. Due to the randomized order of the intervention, and to investigate the within-subject change, the tests were run separately depending on the presenting order (i.e., APA training first or AC training first). The reported effect sizes were calculated (*r*)^3^.

Self-reported safety and efficacy scores of the strategy, gathered during follow-up calls, were analysed and presented using descriptive statistics.

### Content analysis of retrospective recollections

We conducted a direct content analysis (25) on the data resulting from the open questions. Texts based on each testing phase were reviewed by 2 researchers (WY and ZW) to identify key categories that aligned with known psychological constructs underpinning motor control and attentional processing. A third researcher (YR) then reviewed the data inductively to ensure all ideas were captured. The context of each category was reviewed independently by the researchers to identify whether it was used positively, negatively, or mixed, labelled neutral, with resulting consensus (examples are provided in Appendix S1). When the same category was used in the same context several times by a participant this was counted only once. Subsequently, researchers quantified the frequency of positive, negative, and neutral expressions of the key words/phrases across the different testing phases. Finally, the categories were grouped into categories capturing related ideas.

## Results

**Fig. 2:**
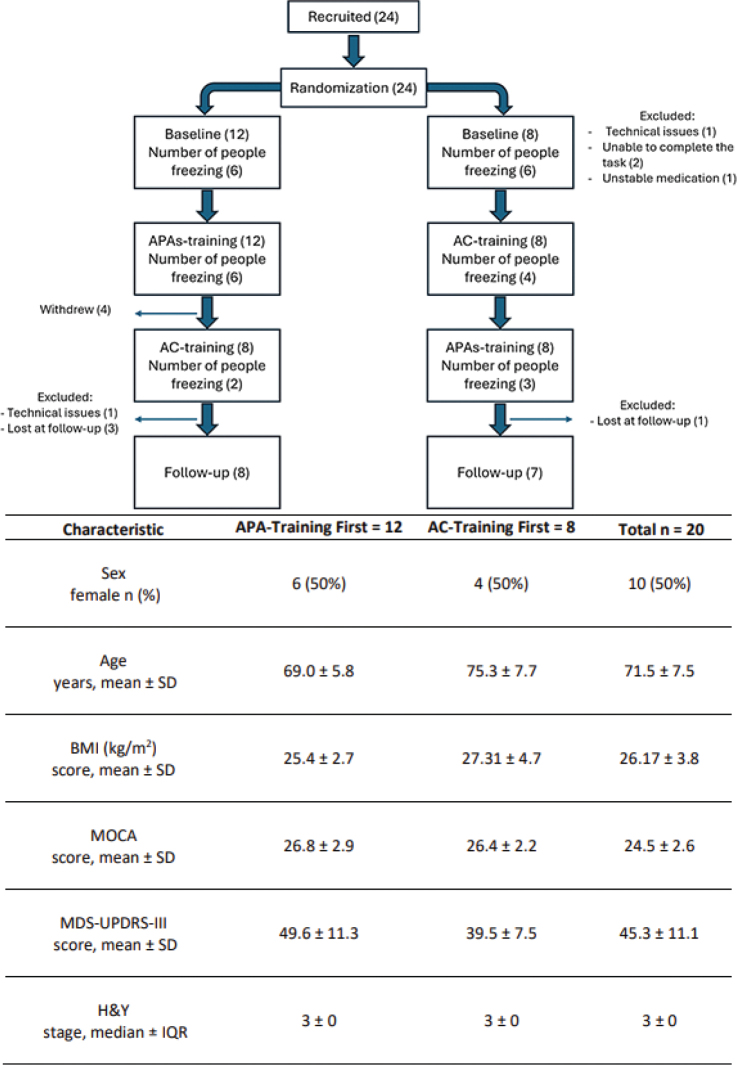
**Participant allocation into the 2 groups and participants’ characteristics.** Top: Participants’ flow chart. The diagram summarizes how participants were allocated to each group, how many completed each phase, and how many experienced FoG in each phase. Bottom: Summarizes participants’ characteristics. BMI = body mass index; MOCA = Montreal Cognitive Assessment Scale; MDS-UPDRS – III = Movement Disorders Society Unified Parkinson’s Disease Rating Scale; H&Y = Hoehn and Yahr Stage; SD = standard deviation; IQR = interquartile range.

Nineteen pwPD were included in the final analysis. Four pwPD were excluded after completing the baseline phase; 1 participant was excluded during data analysis due to technical issues with the video recording system. In addition, 4 participants did not complete the post-AC training phase due to fatigue (Fig. 2).

For baseline, post-APA training, and post-AC training we recorded 200 (2.16 freezes per min of walking), 103 (1.35 freezes per min of walking) and 83 (2.34 freezes per min of walking) FoG events, respectively. Seven participants did not experience FoG in any phase and 1 experienced FoG in baseline phase only. Six participants experienced FoG in both baseline and post-APA-training phase and 4 in both baseline and post-AC training. Participant characteristics are summarized in Fig. 2.

### Effect of the APA intervention on FoG characteristics

The percentage of successful steps from FoG was significantly higher in the post-APA-training (median ± interquartile range = 76% ± 54%) phase compared with baseline (57% ± 77%; Z(5) = –1.826, *p* = 0.034, *r* = 0.69). No significant differences were found in FoG duration, incidence or percentage of time spent freezing between Baseline and APA-training (*p *> 0.05, see Table I in Appendix S1 for more information).

**Table I:** Summary of results from summative content analysis, indicating number of people discussing identified categories in each phase

	Baseline				Post-APA training				Post-AC training			
Participants	Total: 19				Total:18 (Order 1: 10, Order 2: 8)				Total:13 (Order 1: 8, Order 2: 5)			
General strategy	6		DNS		2 (2,0)		DNS		4 (4,0)		DNS	
Action/goal directed	3		10		14 (8,6)		2 (0,2)		3 (1,2)		6 (3,3)	
	Positive	Negative	Neutral	DNS	Positive	Negative	Neutral	DNS	Positive	Negative	Neutral	DNS
Fear & control	0	0	1	18	7 (4,3)	1(1,0)	0	10 (5,5)	1 (1,0)	1 (0,1)	0	11 (7,4)
Worries & distraction	0	4	4	11	2 (2,0)	2(2,0)	0	14 (6,8)	1 (1,0)	1 (1,0)	1(1,0)	10 (5,5)
Outcomes	0	3	0	16	7 (4,3)	0	1 (0,1)	10 (6,4)	1 (1,0)	1 (1,0)	0	11 (6,5)

Numbers in parentheses break down the total number of comments based on the order in which the intervention was presented to the participant (Order 1, Order 2). DNS reports how many people did not provide any comments on a given category.

### Self-reported psychological factors: within-subject comparisons of baseline vs single intervention

When the APA training was presented first (*n* = 11), we observed an increase in confidence from baseline to post-APA training (Z(10) = 2.552, *p* = 0.011, *r* = 0.81), along with significant reductions in both fear (Z(10) = 2.572, *p* = 0.010, *r* = 0.81) and worrisome thoughts (Z(10) = 2.555, *p* = 0.011, *r* = 0.81).

When AC training was presented first (*n* = 8), we observed a reduction in fear from baseline to post-AC training (Z(7) = 2.032 *p* = 0.042, *r* = 0.72); no differences were observed for confidence (Z(7) = 1.841, *p* = 0.066, *r* = 0.75) or worrisome thoughts (Z(7) = 1.134, *p = *0.257, *r* = 0.43)

### Self-reported psychological factors: within-subject comparisons of single vs combined intervention

**Fig. 3:**
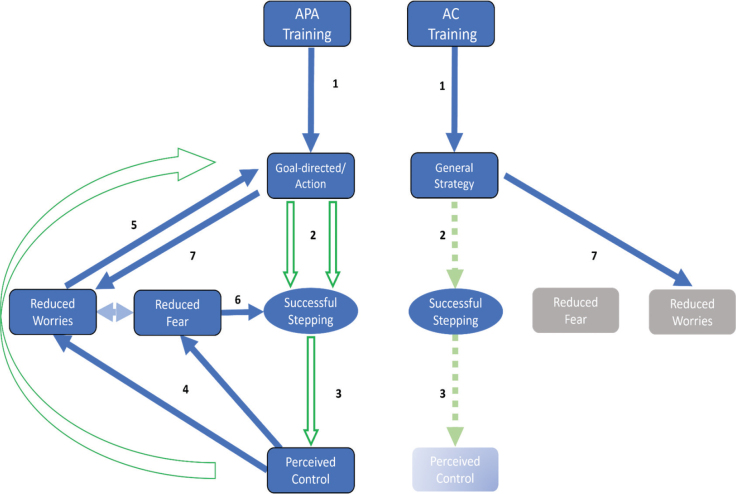
**Conceptual framework highlighting constructs that appear to be modified by the interventions.** The model also highlights potential temporal links between the constructs based on data reported here (green hollow arrows), or those derived from existing literature (blue solid arrows). Faded/dashed arrows represent potential relationships with weak evidence. The training produces the adoption of a strategy (either goal-directed or general). - The strategy increases the likelihood of producing successful steps through psychological and/or biomechanical mechanisms. In the APA training the mechanisms involved are both psychological and biomechanical, whereas in the AC training any changes are presumed to be psychological only. Note that the construct Successful Stepping here represents a combination of: (*i*) observations of effective gait initiation; and (*ii*) verbal descriptions of improved walking ability identified within content analysis. - The increased percentage of successful stepping induced by the adopted strategy increases one’s sense of control. For this content analysis, we adopted the following definition of perceived control: one’s perceived capacity to be able to cope and reach goals under stress (26). - The increase in perceived control reduces perceived magnitude of both fear and worrisome thoughts, in addition to reinforcing the sense of efficacy within the goal-directed/action strategy. - The reduction in distractions/worrisome thoughts frees up cognitive capacity that can be used to engage the action strategy, while simultaneously reducing perceived fear. - Based on the presumed association between arousal and FoG (16), it is likely that reductions in fear will serve to reduce FoG severity, which in turn further improves the likelihood of successful steps. - Having a strategy in place reduces people’s worrisome thoughts/distractions by giving them something to focus on and improving their sense of agency.

Confidence in successfully stepping from a freeze at first attempt significantly increased from baseline (70% ± 38%), to post-APA training (90% ± 21%; Z(5) = 2.032, *p* = 0.021, *r* = 0.77). Additionally, confidence in stepping from FoG without falling significantly increased from baseline (80% ± 25%) to post-APA (92.50% ± 20%; Z(5) = 1.841, *p* = 0.033, *r* = 0.70). In the post-AC phase participants reported confidence levels of 75% ± 25% for stepping from a freeze at first attempt and 90% ± 15% for stepping out of FoG without falling (Appendix S1; Figs 3 and 4).

When APA training was presented first, we observed no differences between APA training and AC training in confidence (Z(3) = 0.000, *p* = 1.0, *r* = 0.00), fear (Z(4) = 1.069, *p* = 0.285, *r* = 0.48) or worrisome thoughts (Z(4) = 1.000, *p* = 0.317, *r* = 0.45). Similarly, when the AC training was presented first, there were no changes in fear (Z(7) = 1.761 *p* = 0.078, *r* = 0.62) or worrisome thoughts (Z(6) = 1.604, *p* = 0.109, *r* = 0.61). However, there was a significant difference in perceived confidence in stepping from a freeze (Z(6) = 2.041, *p* = 0.041, *r* = 0.77), indicating that APA training provided an additional psychological benefit in stepping from a freeze in addition to AC training.

### Follow-up

Of the 19 participants, we successfully arranged follow-up video calls with 15 participants. Three could not recall specific details of either training. Two participants were not able to recall details of the AC training. Twelve participants recalled details of the APA training and reported that they perceived the proposed training as safe (mean score ± standard deviation; 8.7 ± 1.1; maximum = 11) and effective at helping them step from FoG when used in daily life following the laboratory session (7.5 ± 2.3; maximum = 11). All 12 participants who recalled the APA training reported using the strategy most of the time when experiencing FoG. More details about the follow-up questionnaire are provided in Appendix S1.

### Content analysis

Four separate categories were identified in the content analysis: (*i*) strategy, (*ii*) fear & control, (*iii*) worrisome thoughts & distractions, and (*iv*) outcomes. Strategy encompassed 2 sub-categories: general and action/goal-directed strategies. General strategy related to self-instruction that was not specific to any particular thought process or movement sequence (e.g., concentrating on the task, or trying not to freeze). Action/goal-directed strategy related to intended movements and related thought-processes (e.g., weight-shifting). Fear & control encompassed statements regarding participants’ emotional responses to perceived threats, physical tension relating to fear, and one’s sense of control over their thoughts and/or movements. Worrisome thoughts & distractions related to a preoccupation of participants’ thoughts towards things that were not directly related to the walking task (e.g., falling or reporting being distracted by task-irrelevant visual features of the VR environment, such as pictures on the wall). Outcomes gathered statements related to perceived consequences of psychological or physical factors (e.g., walking more fluently due to having greater confidence).

Frequencies of positive, negative and neutral categories across the different phases are reported in Table I, whereas example quotes for each category are reported in Appendix S1.

## DISCUSSION

A single session of independent interaction with video-based education can deliver improvements in step initiation from FoG and associated levels of confidence and reductions in fear and worries.

### Effect of APA training on step success and psychological factors

When comparing the magnitude of the training effect on the percentage of successful vs unsuccessful steps during FoG, we observed slightly smaller effects than previously reported (13) (i.e., medium-to-large vs large). This difference is likely due to the increased task complexity and the use of a video-based training format. However, the observation that most participants deemed the APA training to be safe, effective, and continued to use the strategy regularly in their daily lives underscores its practical utility. The sample size may limit the interpretation of how cognitive deficits impact the effectiveness of this self-generated strategy. Our impression is that the most significant cognitive barrier relates to the initial selection of the strategy during FoG, as opposed to the execution of the strategy once recalled. Greater spontaneity in recalling and executing the strategy is likely to require extended practice and/or support from others. Work is currently under way to evaluate similar outcomes following extended practice with the option of family support and to identify whether potential benefits are achieved across FoG phenotypes and sub-types.

We observed significant changes in self-reported psychological factors following APA -training but not AC training. This provides an initial indication that a single session of video-based APA training has the potential to produce acute reductions in fear and anxiety relating to FoG, despite the content of the education focusing on intended movement outcomes rather than managing psychological factors. Sense of control (26) is a key element of both confidence and worry, which likely reflects the positive changes seen in this practical intervention.

The exact pathogenesis of FoG remains unclear, but scientific evidence suggests it may be caused by abnormalities in: (*i*) central drive/automatic control, (*ii*) central gait pattern generators, (*iii*) perceptual processing, (*iv*) cognitive functions, and (v) the coupling between posture and gait (1, 27, 28). These initial data on APA training may suggest that, due to its ability to target both biomechanical and psychological factors, this strategy may influence several of these elements simultaneously, potentially explaining its overall efficacy in helping people step successfully out of a freeze. Specifically, we speculate that the design of APA training may impact both the coupling of posture and gait, as well as central drive/automatic control, by overriding the inefficiencies in these processes and shifting towards a more conscious control strategy. Additionally, the APA training may influence cognitive functions through the observed increased sense of control. As anxiety is known to pre-empt cognitive resources, the heightened sense of control induced by the APA training may free up cognitive capacity, allowing these resources to be reinvested in producing and controlling movements (17, 18). It is important to note, however, that as the APA training may require cognitive resources to be enacted, this strategy may be less suitable for individuals with more severe cognitive decline.

Given the current data, it is difficult to interpret the extent to which benefits of the APA training are primarily driven by biomechanical (13) or psychological factors. Our content analyses of thoughts collected immediately after the task suggest a compounding effect of several distinct, yet complementary, processes. In Fig. 3, we provide an illustrative diagram which highlights factors that appear to be modified by each intervention, in addition to mechanistic links between these factors that can be established either through data reported here or through established theory within the literature.

The most striking observation within the content analysis and Fig. 3 is that the APA training was associated with positive changes in a range of constructs that reflect a beneficial cyclical relationship between the adoption of goal-directed attention, improved ability to step from FoG, increased perceived control over FoG, and reductions in fear and worries. The adoption of goal-directed focus of attention, a clear action-specific movement strategy (Table I), which relies on a conscious generation of a weight-shift action, could be argued as the most critical in terms of the influence on successful gait initiation.

When considering the significant modification in self-reported fear, worries, and confidence relating to FoG following APA training (Table I), it is important that we interpret the meaning of these outcomes beyond their mechanistic influence on FoG and with reference to the lived experience of FoG. FoG pathology is associated with increased prevalence of anxiety and depression (1). Traditional conceptualizations focus on these symptoms being primarily driven by neurobiological changes associated with PD (16, 29). However, they are also likely to be a consequence of perceptions of movement difficulties and the impact that these have on people’s perceived identity and willingness to engage in activities (29, 30). These anxieties appear to be a consequence of uncertainty concerning one’s ability to move as intended and the expectation of negative outcomes. These perceptions and expectations represent a formidable challenge for the development of interventions designed to manage anxieties regarding FoG.

Our data would suggest that providing a cognitive strategy to alleviate anxiety (albeit over only 1 session) is not an effective strategy in this regard. Current interventions designed to manage anxiety in pwPD tend to be multifactorial, incorporating elements of mindfulness and cognitive behavioural therapy. However, evidence of their effectiveness is limited (31) and they tend not to be specific to FoG. In applied contexts, we envisage that APA and AC training should not be considered as standalone interventions. Rather they should form a specific “module” within a broader supported intervention framework incorporating wider range of elements (e.g., coping, self-help [31]), whereby any benefits of the APA/AC training might be capitalized upon and consolidated through the synergistic effects of the broader supported interventions and multiple sessions. Future interventions designed to help people manage anxiety around FoG should include training on “rescue strategies” specifically targeting gait initiation and turning.

### Impact of APA training on FoG characteristics

Most interventions seek to reduce the percentage time in FoG, as it is the most common clinical outcome used to objectively infer FoG severity. Contrary to our original prediction, we observed no significant changes in FoG duration before and after the APA training. However, in reconsidering our initial (pre-registered) hypotheses it must be noted that a self-generated “rescue strategy” that explicitly instructs users to make a conscious attempt to stop and take their time to prepare for, and engage, a learned action sequence will likely inflate time spent in the frozen state. Improving the result of the process is the critical outcome (percentage successful vs unsuccessful step attempts), even if it took some time to produce. It is, however, important to note that these periods of preparation are likely to reduce with more practice, potentially ultimately leading to reductions in freezing duration and frequency.

### Limitations

This study has 3 main limitations. First, although we allowed a substantial familiarization period (~20 min) to minimize order effects, baseline was always completed first. As such, we cannot discount trial-order effects. It is important to note that order effects relating to learning and familiarization are likely to have been counterbalanced by fatigue and/or fluctuations of motor symptoms induced by medications wearing off. Additionally, the familiarization may have contributed to post-intervention changes in mean values of self-reported fear, anxiety, and confidence to some extent. However, it is important to consider that the null results for our active control phase (AC training) indicate that significant effects observed following APA training were not solely a consequence of task familiarization. The absence of within-subject changes in self-reported fear or worries following AC training indicates potential shortcomings in approaches that provide education on anxiety in isolation (32). Second, the low number of participants experiencing FoG at baseline, together with the overall low incidence of FoG events and the withdrawal of 4 participants (due to fatigue) after the APA-training phase (arm 1 of the study), prevented direct comparison of FoG characteristics between the 2 interventions and between groups after exposure to both interventions. Future studies should investigate whether AC training has any effect on FoG characteristics and whether there is any added value in combining the 2 interventions. Third, although efforts were made to simulate a more realistic everyday environment, this study was conducted in a controlled VR setting and did not evaluate the impact of dopaminergic medications on the participants’ ability to use the strategy. Nor did the current study have a sufficient sample to make comparisons between FoG sub-types or phenotypes. Current work is under way to address these shortcomings and to evaluate the efficacy and safety of the video training in community settings and following a longer period of practice.

### Conclusions

This study provides further evidence that APA training is effective in supporting people with Parkinson’s and FoG to step from a freeze in challenging and complex tasks and environments. The observed changes in the percentage of successful steps following a freeze are the result of both biomechanical (i.e., functional APA) and psychological (i.e., increased confidence, reduced worries, and associated increases in goal-directed focus of attention) factors. Further work is necessary to evaluate the efficacy and safety of APA training in community settings and following a longer period of training.

## Supplementary Material



## Footnotes

1 The different scales were employed to clearly differentiate between the constructs of general confidence and thought processes related to fear and worries. On all scales, lower scores indicated the absence of these thoughts (i.e., not at all), while higher scores reflected their more extreme manifestations (i.e., completely/constantly).

2 Steps were considered successful if they enable the wilful continuation of the desired task. Detailed information on the identification of successful vs unsuccessful steps and the criteria used is available in Appendix S1.

3 The effect size (*r*) was calculated as described in Rosenthal R. Parametric measures of effect size. In Cooper H, Hedges LV, editors. The handbook of research synthesis. New York: Russell Sage Foundation; 1994, p. 231–244.
