# Supplementary material for: EFFICACY OF A SINGLE SESSION OF ANTICIPATORY POSTURAL ADJUSTMENTS TRAINING TO SUPPORT PEOPLE WITH PARKINSON’S OVERCOMING FREEZING OF GAIT: A MULTI-METHODS APPROACH
Source: J Rehabil Med. 2025 May 16;57:42491. doi: 10.2340/jrm.v57.42491 (PMC12105535; doi:10.2340/jrm.v57.42491)
Supplement: Supplementary file 1 [file JRM-57-42491-s1.pdf]

Supplementary material has been published as submitted. It has not been copyedited, or typeset by Journal of Rehabilitation Medicine

Appendix S1

SUPPLEMENTARY MATERIAL

Experimental Protocol

Each phase was carried out as follows. After an initial standing period, participants were invited to walk forward until they reached the red circle where they were instructed to stop. Then, they were asked to perform a modified Ziegler's turning task [22]. Briefly, the task involves performing a 360˚ turn in one direction, and then a second 360˚ turn in the opposite direction. We asked participants to make clear stops between the turns (e.g. clockwise turn – stop – counter-clockwise turn – stop). Following the Ziegler's turn, participants were instructed to walk through a doorway, perform a 90˚ turn and keep walking until reaching the following red circle, indicating the beginning of another Ziegler's turn. A graphic representation of the walking task is provided in Figure 1. Additionally, an introductory video of the study is available in the following link (https://www.youtube.com/watch?v=OGstCCAKhig). The video presents the study's main purpose, the laboratory setup and the tasks involved.

The walking tasks and the VR did not change across the 3 phases, but people were asked to behave differently in case of a FoG event. Specifically: i) Baseline. Participants were instructed that if a freeze occurred, they should attempt to try to start walking/turning in the intended direction as they would normally do. ii) Post-APA-Training. When try to start walking/turning during a freeze people should try to stop, shift their weight towards their non-stepping leg and then try to continue the motor task they were doing before FoG occurred (i.e. walking or turning); iii) Post-AC-Training. In case of a freeze, people should try to focus on a self-selected strategy to overcome freezing rather than on the problem they are experiencing. Participants were given 5-10 minutes to practice each strategy before being tested. Due to the nature of the experiment, it was not possible to blind the participants to the phases order.

In each phase, people navigated the VR environment both in clockwise and counterclockwise directions and they were asked to carry on with the tasks until otherwise instructed. For each participant, we aimed to collect data from 6 corridors in each direction, and in each phase (36 corridors in total). However, for some participants data collection was terminated earlier at their request. On average people spent about 4 hours in the laboratory (including pauses).

Identification of successful/unsuccessful steps

FoG has been defined as a "brief, episodic absence or marked reduction of forward progression of the feet despite the intention to walk." (Nutt et al., 2011). In line with this definition, to classify an event as FoG there must be a visually identified failure to make forward progression despite the intent to move. In the annotation

process we utilised in this paper and in two previous studies (Kal et al., 2023, Maslivec et al., 2020) we focussed only on the visually apparent movements deemed to represent an attempted step initiation. We acknowledge that a potential limitation in this approach is with akinetic freezing, in which attempts to step are not readily visually apparent. However, we argue that more common outcomes are also compromised in this context. For example, we observe that, when using movement strategies such as those described in Maslivec et al. and Kal et al. participants may intend to stop and take their time to use the strategy to evoke the weight-shifting. As such, we argue that this period no longer constitutes a freeze, given the apparent absence of attempted forward progression. In this example total freeze duration is artificially inflated by virtue of the participant taking their time to use the rescue strategy.

While annotation of attempted (and unsuccessful) steps adds complexity to a process that is already notoriously challenging, we argue that this produces a richer dataset that is more closely aligned to inferences of a walker's intent to step. This inferred intention to step is already the critical factor that determines outcomes in current annotation processes, following Nutt's et al., definition of FoG. We merely propose to extend these inferences to incidents where there is a visible clear attempt to step that did not result is successful gait initiation. The main confound within this approach relates to incidents where there might be a clear attempt to step, but no visible manifestation of this intent. This is particularly relevant for akinetic FoG. However, we argue that ambiguity in the inference of intent is not limited to our proposed approach but is pervasive across all commonly used forms of subjective video-based annotation (Cockx et al., 2022, https://doi.org/10.1002/mdc3.13556). As such, we suggest that ongoing attempts to evaluate automated approaches to detect FoG onset/offset should also include comparison of unsuccessful attempts to step. These processes would be better informed by studies evaluating inter- and intra-rater reliability of unsuccessful steps.

In the current study, a panel of trained researchers first identified FoG events by watching the video recordings (25Hz) of the walking trials. After the identification of the FoG event, with its onset and offset, the latter described as the continuation of the task (either walking or turning), researchers made categorical judgments about whether the first attempted step following FoG onset was successful or unsuccessful in continuing the task performed. To be considered an unsuccessful step, distinct within the overall FoG event, the step had to meet ALL the following criteria:

- There is a clear separation between the overall FoG event and the attempted step. After freezing, the participant brings the feet back to the floor and the knees in a neutral position. If the feet and/or knees are not brought back in a neutral position, then the FoG event is considered as still ongoing. If the person is able to progress forward without bringing the heel back to the floor, those events are either considered successful steps or shuffling.
- Following the reached neutral position there is a clear and volitional attempt to step. Unsuccessful steps are identified as clear yet incomplete movements of the foot and ankle joint typical of a step (i.e. heel off) that do

not lead to the natural continuation of the task (i.e. heel off and subsequent foot contact). Following this definition, shuffling does not fall within the definition of an attempted step because it lacks the *clear and volitional attempt* components.

- Knee role. The knee joint is evaluated conjunctly with the ankle. Knee movements associated with stepping (i.e. small but clear discrete flexion and forward movement at the knee joints) are considered to be indicators of an ongoing attempt to step. Knee trembling or disorganised knee movements were considered indicators of the FoG event still ongoing.
- All the members of the panel need to agree on the final definition. In case of discrepancies in the definition of the event, members of the panel would discuss openly about the event to find a common agreement. In case there was no unanimous decision upon whether a certain step was either successful or unsuccessful the event was computed in the total number of FoG events, but it was not classified neither as successful nor unsuccessful.

Particular cases:

- Change of the leading leg. In long attempts, some participants tend to try to step with one leg before changing the leading leg to carry on with their task (e.g. try to initiate walking with the right leg but after some unsuccessful attempts they step with the left one). In this case, the first attempted step was considered as being unsuccessful.

Training Videos

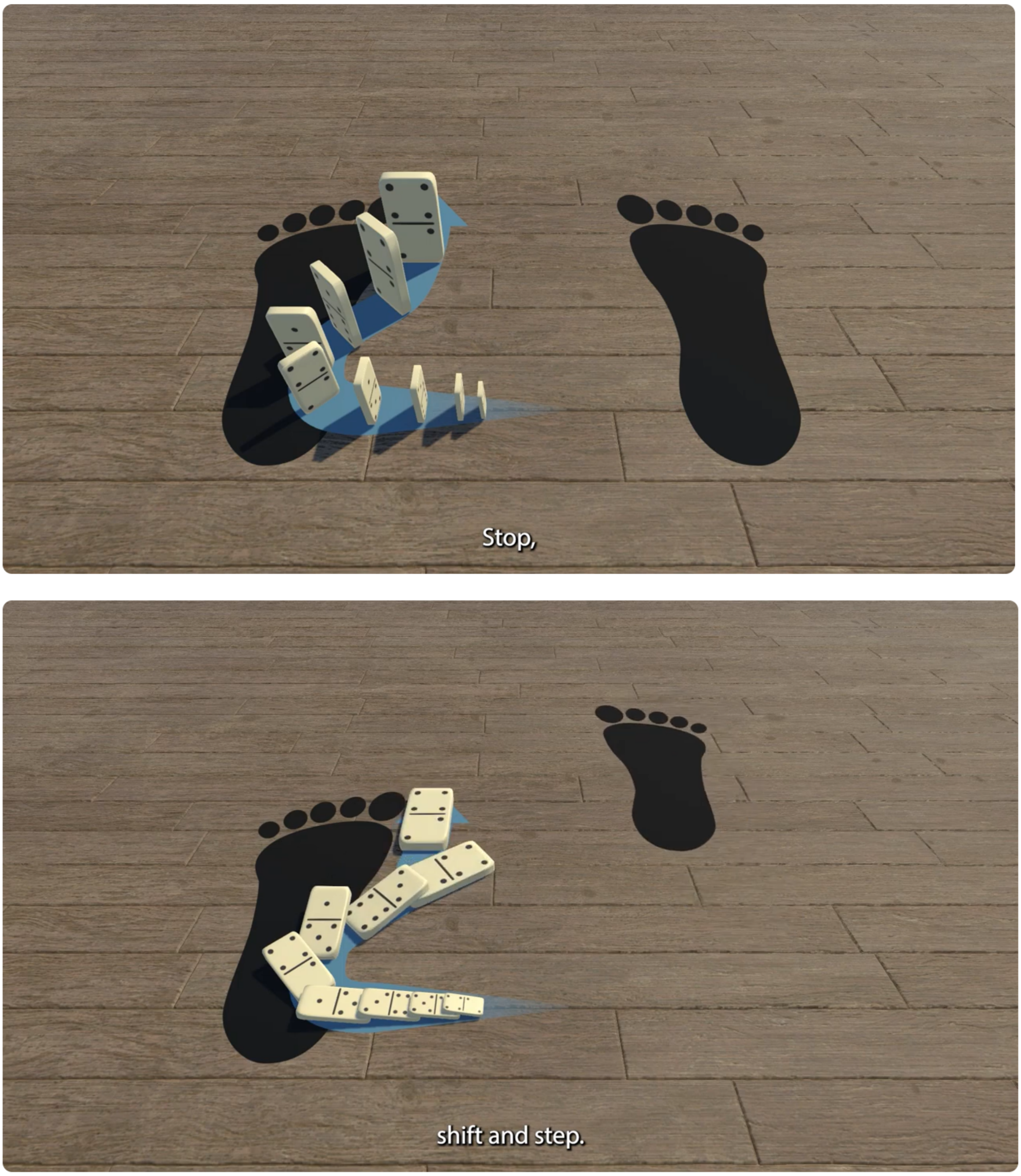

Figure 1. *The top and bottom figures depict scenes from the APA-training videos. These specific frames illustrate the analogy of taking a step as the result of a domino effect, where the last domino (i.e., taking a step) only falls if preceded by a series of events (i.e., stop and the weight shift).*

The APA-training video begins with a brief explanation of FoG, incorporating the experiences of people with Parkinson's disease. It then provides a detailed description of the APA strategy, using examples and analogies to demonstrate how it can be applied during both straight walking and turning. The training also emphasizes the importance of safety when practicing the strategy, offering suggestions to minimize the risk of falling. Finally, it introduces the next video, which covers the AC-training.

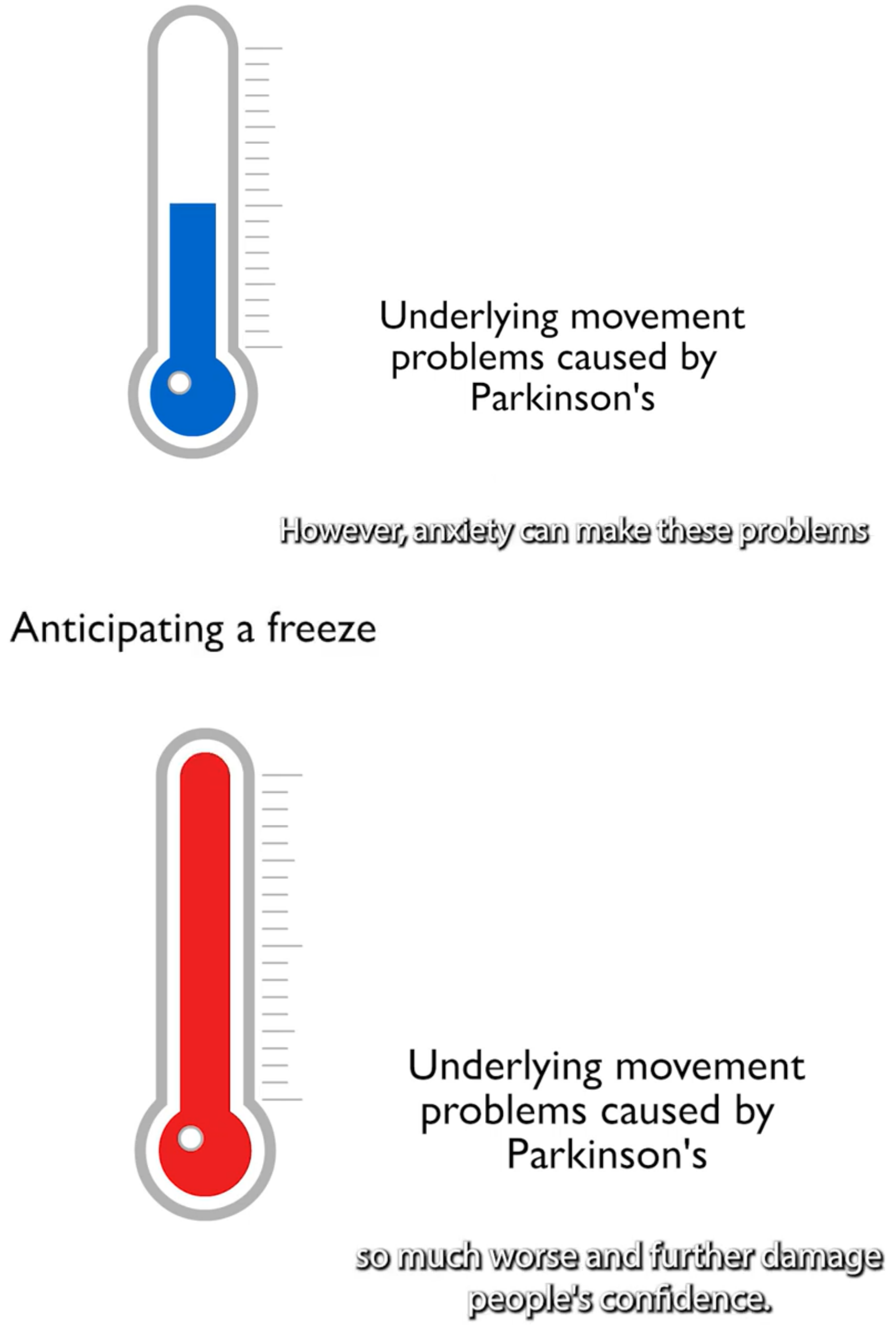

Figure 2. *The top and bottom figures depict scenes from the AC-training videos, using the analogy of a thermometer. Our system has a limited capacity to handle cognitive demands, and when factors such as anxiety and worries absorb additional cognitive resources, the "thermometer" may break—representing further impairment of motor symptoms and an increased occurrence of freezing of gait (FoG).*

The AC-training video begins by outlining its aim, introducing the negative processes that can exacerbate the incidence and duration of FoG. Individuals with Parkinson's then share their experiences and emotions, providing practical examples of how these negative processes can worsen FoG. The training highlights the importance of shifting focus from the problem (FoG) to the solution (safely taking a step), offering practical strategies to manage the negative emotions that contribute to worsening FoG.

4- Weeks Follow-up Questions

1) Have you used the strategies? (Open response)
2) Have you found it/them useful? (1-11 scale not at all - very useful)
3) How often do you use the strategies? (frequency per week and % of times frozen)
4) Do you use the strategies more/less in particular situations/places? Also ask for a reason for the answer (Open response)
5) How do you use the strategies? What do you think about? And does this change at all? (Open answer)
6) Have you practiced the strategy? If so, how? And how often?
7) Do you feel safe/unsafe using the strategy? (1-11 very unsafe - very safe)
8) Do you feel like weight-shifting is more or less stable compared to your alternative way to start walking? (Open response?)
9) Have you been using this strategy with a partner/family/friend giving you a prompt? (Yes/no) If so, how have you been doing this? (Open response)

Additional Figures

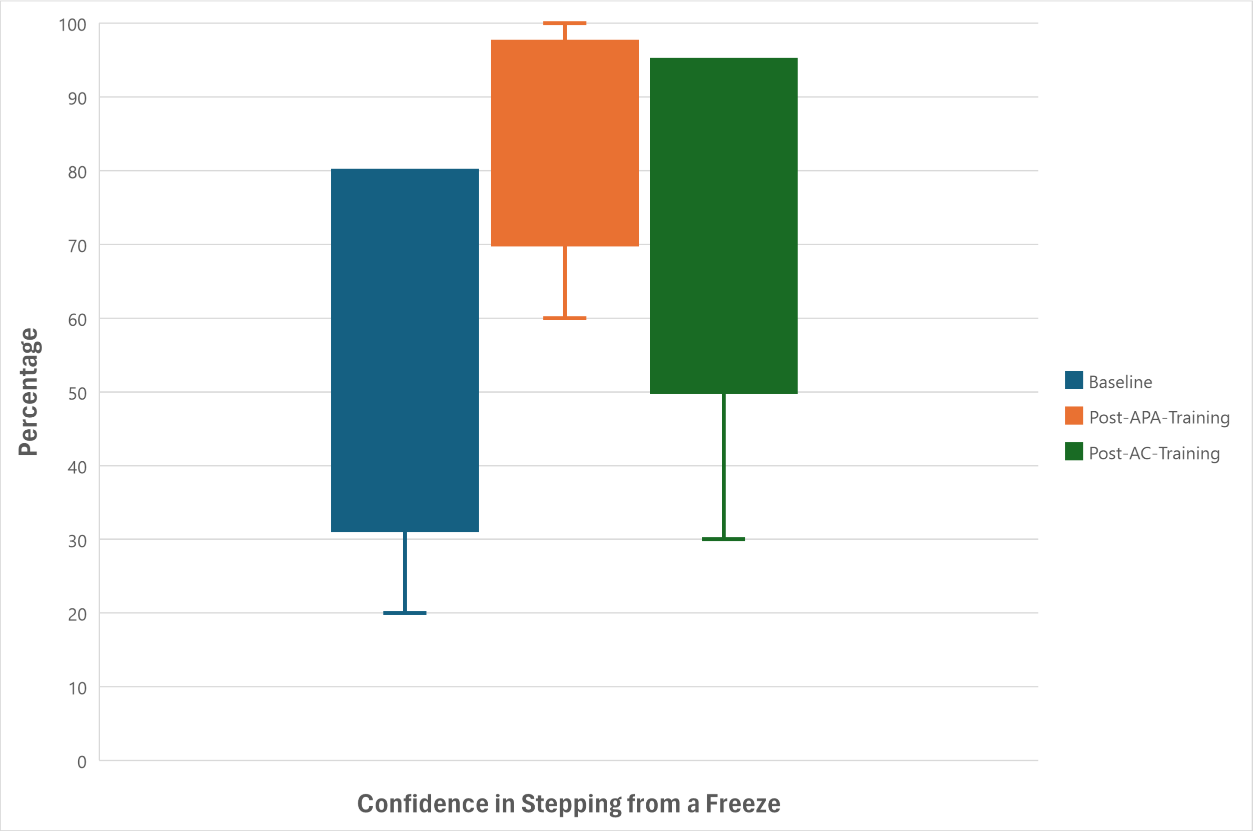

Figure 3. *Self-reported confidence in stepping out of a freeze without falling, measured across the different experimental phases. Please note that the absence of error bars in some boxplots is due to the small distribution of the data likely caused by the limited sample size.*

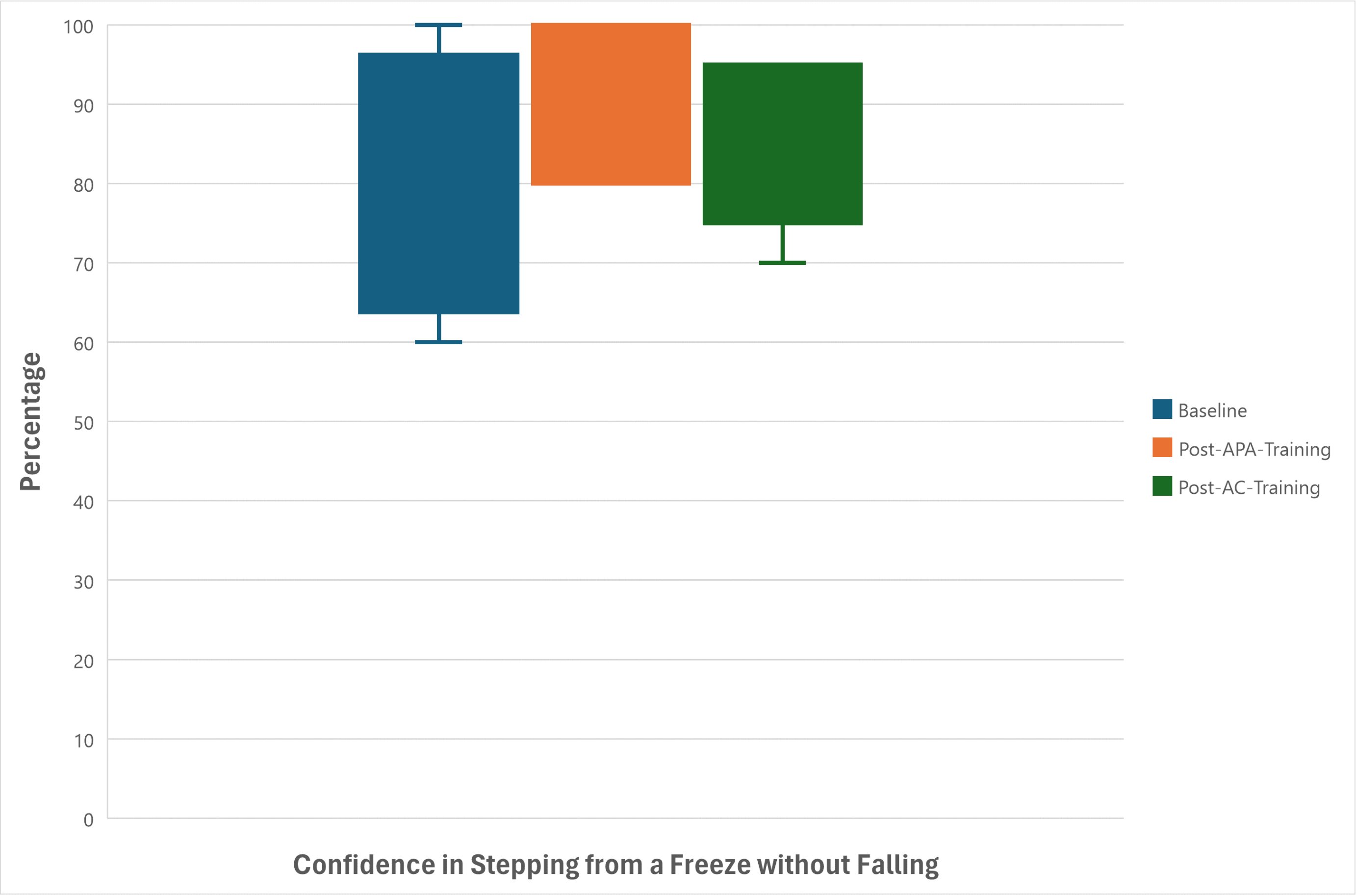

Figure 4. *Self-reported confidence in stepping out of a freeze without falling, measured across the different experimental phases. Please note that the absence of error bars in some boxplots is due to the small distribution of the data likely caused by the limited sample size.*

Variable	Z Score	Degrees of Freedom	P Value	Effect Size (r)
Number of FoG events	1.473	5	0.141	0.60
Mean Duration of FoG events	0.105	5	0.917	0.04
Percentage of Time Spent Freezing	1.363	5	0.173	0.56

Table 1. *Statistical results for FoG outcomes between the Baseline and post-APA phases.*

Themes & Subthemes	Example Quotes
Strategy General	"Walk with intention, head for where I was going."
Strategy Goal-directed/Action	"Use Stop Shift Step as an extension of positive thinking. Using WS as a trigger to get positive things going. Using less effort. I used it halfway through the turn to keep momentum. Just a small movement enough to get benefits."
Fear & Control	
Positive	"proving myself more into the unknown. I shuffled once and as soon as I knew I could do it I felt more confident, I found this more relaxing. I started to enjoy the movement too. I could visualise it in a positive way. It was a complete surprise."
Negative	"I was having a crisis of confidence feeling like I was coming off."
Neutral	"The fear, if you can't control it, you cannot control anything. It is engulfing me, controlling the fear. The feeling of nerves stops you in your tracks. If you do something that works well, your situation gets better."
Worries & Distractions	
Positive	"I was less confused about stuff somehow ...it just didn't worry me."
Negative	"I was getting a bit distracted. "
Neutral	"I was trying to ignore the pics, bags and doorway."
Outcomes	
Positive	"Felt quite good actually. Made me feel as though I was walking slightly more fluently."
Negative	"Felt hesitation in my body, I was aware that it was happening"
Neutral	"No more or less difficult."

Table 2. This table offers example quotes corresponding to each category and sub-category.
